# The meaning of reflection for understanding caring and becoming a caring nurse

**DOI:** 10.1111/scs.13080

**Published:** 2022-04-01

**Authors:** Turid Anita Jaastad, Venke Ueland, Camilla Koskinen

**Affiliations:** ^1^ Department of Caring Science Faculty of Education and Welfare Studies Åbo Akademi University Vaasa Finland; ^2^ 56627 Department of Caring and Ethics Faculty of Health Sciences University of Stavanger Stavanger Norway

**Keywords:** becoming, caring, nurse education, reflection, student nurse

## Abstract

**Background:**

Reflection is essential for students to learn and understand caring, their formation as human and caring beings, and their ability to meet patients in a caring way. Consequently, to facilitate nurse students’ development into professionals, learning support is needed where the focus is on understanding caring and becoming caring nurses.

**Aim and research questions:**

The exploratory study aim is to gain knowledge of the meaning of reflection in first‐term nursing education, and how reflection grounded in caring theory can deepen the students’ understanding of caring and their professional formation of becoming a caring nurse.

**Method:**

Data consisted of individual written reflections and were collected from 64 nursing students from Norway, who had completed their instruction in caring theories and participated in four reflection groups where they reflected on caring and becoming a caring nurse. A thematic analysis was used.

**Findings:**

The results are based on the three main themes, Reflection provides an understanding of caring by developing a language for caring; Reflection provides an understanding of seeing the person behind the illness; and Reflection contributes to increased self‐understanding and awareness of oneself as a caring nurse.

**Conclusions:**

Instruction in caring theories and participation in reflection groups, with reflection grounded in caring theory, has a key function in facilitating students’ development of a language for caring in nursing and appropriation of caring theory. The appropriation of caring theory provides a foundation for the nurse students to see themselves within a broader perspective and is important for mutual support in the professional formation of becoming a caring nurse. The expected outcome of such integration is a nursing curriculum that progressively supports the development of nursing students professionally and personally in the formation of becoming a caring nurses.

## INTRODUCTION

The starting point for this study is the challenge in nursing education to create fruitful conditions for nurse students’ learning and reflective processes that strengthen their understanding of caring and their professional formation to become caring nurses [1, 2, 3, 4]. Essential to the process of becoming a caring nurse is the appropriation of a caring attitude where the nurse students, through their actions and attitudes, alleviate human suffering and meet the patient with dignity and compassion [5, 6]. The current study is rooted in caring science education with a belief in students’ ability to develop and form in learning. This implies a holistic formation and molding, educating caregivers with a caritative ethical attitude and professional competence [7].

Caring is widely described by nursing and caring theorists as an important topic and a core mission, and as an ethical value for nursing professions [8, 9, 10]. Suárez‐Baquero et al. [11] highlight that in the art of caring is embedded the core disciplinary nursing knowledge. An interesting starting point is whether a human being by nature is caring or if caring can be learned and trained. According to Eriksson [12], human beings are inherently caring, while for Olshansky [13] and Boykin & Schoenhofer [14], caring is not necessarily an innate trait in nurses, but more a process and an outcome that occurs over time and involves mastering caring behaviour. Several studies support the idea that caring can be learned and emphasise that the core aim of nursing education is to develop nursing students’ caring competencies [15, 16, 17]. Among many recommendations, nursing education is suggested to focus on strengthening the caring identity of students, enabling them to match their idealistic caring vision with that agreed upon by the nursing profession [18, 19, 20]. Other recommendations are to facilitate the students’ reflection on their experience of caring in personal life and clinical reality [21]. According to Hörberg [22], true caring is not possible without carers adopting a caring attitude to gain insight into the patient's lifeworld and thus an opportunity to understand the patient's lived experiences and life situation. A wealth of literature demonstrates that the students’ awareness of the reflective process within themselves, reflection interacting with caring science theory, can integrate and improve the ability to care for suffering human beings [23, 24, 25, 26, 27, 28]. Sandvik et al. [29] similarly indicates that through active and continuous reflection, caring science theory can live and become lasting.

As reviewed above, previous research shows that the use of caring science theory as part of the reflective process is seen as essential for students’ learning and understanding of caring, their formation as humane and caring beings, and their ability to meet the patient in a caring way. Hence, we find that reflection related to caring theory and students’ unfolding understanding of caring has not been fully explored.

Although reflection is considered a significant component of nursing education and practice [30], the literature does not provide a consensual definition of or model for it [31]. This study is grounded in both phenomenological and hermeneutic views on learning and reflection. Within the phenomenological tradition of caring science didactics, the platform for learning and reflection is the life‐world [32]. According to the phenomenology of Edmund Husserl, the life‐world is related to a natural attitude and involves a type of approach to our everyday activities [33]. The natural attitude characterises activity which humans are completely directed towards, immersed in, absorbed by, or of being in the moment. In everyday activities, humans do not consciously analyze but take for granted what they experience or what they are absorbed by, as existing the way they perceive it. Consequently, a person's natural attitude is basically unreflective. All meaning has its origin in the life‐world which is the prerequisite for all cultivation of knowledge. The life‐world can, according to Husserl be examined and conceptualised through reflection. Through reflection, phenomena of the world will be brought to awareness and so made available for analysis, instead of being taken for granted. In this way phenomena in the lifeworld might be conceptualised and articulated, which is the basis of the learning process [34, 35]. Through a holistic approach to the connection between learning and caring, and based on a life‐world perspective, earlier research within caring science didactics has shown that reflection and the interaction between caring and learning can improve both caring and learning in caring contexts [36].

From a hermeneutical perspective of caring science didactics and according to Gadamer [37], learning is related to an individual's understanding process. Understanding always occurs as a fusion of contemporary and historical horizons. The contemporary horizon is always constituted from the past. It is always possible, according to Gadamer, to open and broaden the contemporary horizon, and even necessary for the development of new understanding [37]. From that point of view, reflection arises in the encounter between experience and pre‐understanding. The reflective activity leads to a new understanding and Gadamer describes this phenomenon as a ‘horizon fusion’ [38]. Reflection is connected to the processes of understanding and becoming. Understanding and becoming are ongoing processes of appropriation, thus transforming students both professionally and personally. Through appropriation, a new understanding is incorporated into oneself that alters oneself, thus changing one`s thought and action, doing and being [2], and the appropriated caring science theory provides authority and courage to practice nursing care [29].

### Aim and research questions

The exploratory study aim is to gain knowledge of the meaning of reflection in first‐term nursing education, and how reflection grounded in caring theory can deepen the students’ understanding of caring and their professional formation of becoming a caring nurse. The research question is: How does reflection ground in caring theories deepen nurse students understanding of caring and of themselves as caring nurses?

## METHODS

### Setting and participants

The study was carried out in 2019 at a Norwegian university. All 330 students in the first‐term undergraduate nursing program were invited to participate in the study: 64 agreed to participate, 48 females and 6 males. The sampling and data collection took place before clinical practice.

Reflection seminars focusing on Kari Martinsen's [10], and Katie Eriksson's [12] theories about caring that are implemented in the undergraduate nursing program in the first term. The students met four times in groups consisting of 10‒12 students in each group. The caring concepts used in the actual education were inter alia, caring communion, caring relationship, natural caring, compassion, kindness, and the fundamental idea to alleviate suffering and promote health and life.

### Data collection

Individually written self‐reflections were used for the data collection [39] and provided the empirical data material for this study. After the four reflection seminars participants were asked to write individual self‐reflection related to the following questions: *What has reflection meant to you? What new insights has reflection contributed to in relation to caring and about yourself as a caring nurse?* The reflection questions were open and related to the students’ thinking and understanding of caring after the first term of their nursing education. The purpose was not to investigate what the students had learned about caring and caring theory but to gain knowledge about the students’ own reflections in relation to themselves in becoming caring nurses. The material consists of 44 typed pages. The written reflections were not lengthy but had rich units of meaning. The students used their own words and they reflected on their own experiences and understanding of caring and nursing. An example of a student reflection:

The theory has helped to concretise and understand the concept of care. Knowing in more detail what is included in the concept from a theoretical perspective provides a more holistic understanding. This will help to reflect more effectively on the topic later. To remember and use something in daily life as a nurse, one must first have understood it properly. Linking theory to reflection in plenary as we have now done has been a great help in understanding caring. Caring is extremely important in a nursing situation and being able to put into words the theory and things I will experience in practice will be useful.

### Analysis

The reflection notes were analyzed with thematic analysis in six steps according to Braun and Clarke [40]. All authors read the reflection notes, and a first analysis was performed. This formed the basis for the preliminary analysis that was conducted by one of the authors (TAJ) Then, all authors convened to discuss the patterns until a unanimous interpretation was agreed upon. To improve trustworthiness and enable readers to follow the research process, the authors strived to describe it as clearly as possible. The first step was to become acquainted with the data. Each note was read several times and notes about ideas were taken. The second step was initial coding. In this step, interesting features among participants’ statements were coded thoroughly and in accordance with the whole data. In this way, relevant data appeared. The third step was to look for themes. In this step, similar codes put together resulted in initial themes. The fourth step was reviewing these themes. In this step, the coordination between codes, related initial themes, and the entire data set was confirmed. In this way, a thematic map was generated. The fifth step was the definition and naming of extracted themes. In this step, the entire process of analyzing was reviewed to refine the characteristics of each code and the story which the whole analyzing process tells. The sixth and last step was reporting, we returned to the research question, and the results were described using themes and sub‐themes, and quotes from the interviews were used to strengthen the credibility of the themes and enable readers to evaluate the transferability of the findings [40]. Based on the research questions and thematic analysis, three themes and nine sub‐themes are highlighted as follows (See Table 1. Overview of findings).

**TABLE 1:** Overview of findings

Themes	Subthemes
Reflection provides an understanding of caring by developing a language for caring	Reflection provides a deeper understanding of caring as a basis for nursing Reflection facilitates understanding and concretisation of caring theory Reflection provides a language for communicating caring
Reflection provides an understanding of seeing and meeting the person behind the illness	Reflection provides a deeper understanding of the patient's perspective Reflection provides an understanding of the importance of meeting the patient as a complex, whole human being Reflection provides a greater awareness of the importance nurses’ caring has for the patient
Reflection contributes to increased self‐understanding and awareness of oneself as a caring nurse	Reflection gives the opportunity to get to know oneself better Reflection provides an increased awareness in assessing oneself Reflection gives new thoughts about one's desire to meet the patient in a caring way as a nurse

### Ethical approval

This study, conducted in 2019, conformed to the principles outlined in the Declaration of Helsinki (World Medical Association Declaration, 2005) and permission to recruit the participant was sought in relation to the Norwegian Centre for Research Data (NSD). Permission was granted by the management of the university. The informants gave written permission for their participation in the study. The research participants were informed of the aims and purposes of the research and collaboration was voluntary and anonymous. They were also informed of the right to refuse to cooperate [41] and that the collaboration in the research is nonbinding and does not affect the final assessment of the study. The participants had an opportunity to withdraw at any time without any explanation. The reflection notes were sent as a word document by e‐mail and given a code. Names and code lists were stored in paper form in a locked drawer in a locked office, separate from the reflection notes. E‐mails with reflection note attachments were deleted.

Individually written reflections were an appropriate approach to this topic to promote knowledge of how reflection grounded in caring theory deepens the students’ understanding of caring and of themselves in becoming caring nurses. We chose to invite all students. There is always a risk that the students who volunteer may have a special interest and positive view of the topic, and we were aware that the text has been shaped by the students writing something that they know will be read by the researcher.

## RESULTS

The description of the results is based on the three main themes, *Reflection provides an understanding of caring by developing a language for caring*; *Reflection provides an understanding of seeing the person behind the illness*; and *Reflection contributes to increased self*‐*understanding* and *awareness of oneself as a caring nurse*.

### Reflection provides an understanding of caring by developing a language for caring

The results show that reflection provides a deeper understanding of caring as a basis for nursing and for becoming a caring nurse. This discovery provides new insights and a deeper understanding of the importance of caring in nursing. *I have become more aware that this is perhaps the biggest role of being a nurse*. Reflection on themes of caring theory opens for the students to recognise that caring in nursing is based not only on a natural caring attitude toward other people but that caring in nursing also requires that the nurse students learn to change both their being and action. *Caring is something all people can provide*, *but as a nurse we need some more professional knowledge*. In the reflection, they discover that caring theory can be a resource in their own learning process towards ethical thinking and formation to become a caring nurse. *Caring for someone you know comes automatically for most people but caring for a stranger does not necessarily come by itself*. *For some it must be learned*, *and then the theory of caring will be very useful*. Thus, the caring theory comes alive and inspires learning and professional and personal formation to becoming a caring nurse.

Reflection facilitates the understanding and concretisation of caring theory. Through reflecting with others, the concept of caring and caring theory become more concrete and understandable. *Reflection has made it easier to understand heavy theoretical concepts and questions*, *and use them in practical examples so that it has become easier to understand*. The students discovered that words and concepts can have a deeper meaning and significance. In the reflection, the students help each other to concretise the caring theory, and manage in a new way, both to understand how they can take care of the patient in a caring way, and how important caring is for patients and relatives.

Reflection provides a language for communicating caring. All the students have their own understanding of the concept of caring, but it is difficult for them to describe the content of the concept in their own words. In dialogue with fellow students, they gain access to their already implied understanding, and the language opens for them to be able to understand and describe caring in other and new ways. *Now I can better put my thoughts into words*, *and I have a better opportunity to communicate about this*. In the meeting between one's own and others’ thoughts a new understanding is formed, and the dedication of caring theory makes it easier to articulate and verbalise caring in nursing.

### Reflection provides an understanding of seeing and meeting the person behind the illness

Reflection provides a deeper understanding of the patient's perspective. In the reflection, the students become more aware of how human beings may experience becoming patients. Through reflection, students develop a deeper understanding of the patient's vulnerability and uniqueness. *I have thoughts about being a patient*, *that you are then in a very vulnerable situation… and that everyone's story is unique*. They also develop a deeper understanding of human vulnerability and interdependence with other people. In the reflection, the students gain a deeper awareness that their own vulnerability can be a strength for understanding the patient's vulnerability. *I have gained a better understanding that as a nurse it is important to be vulnerable*, *to be able to show compassion in the best possible way*. When the students intertwine the caring theory with their own past experiences and pre‐understanding the caring theory becomes more personal.

Reflection provides an understanding of the importance of viewing the patient as a complex whole human being. The students discover the significance of developing an open and responsive approach to the patient, where they show respect and humility for the patient's life‐world. *I have learned a lot of new things*, *how to meet the patient*, *and how to treat the patient as a person*, *and not just the disease or diagnosis the patient is admitted for*. In the reflection, the humanistic views of human beings and the main values that define professional nursing become clearer, and the students gain a deeper insight into caring as meeting the patient as a unique and whole person. In the reflection, the students share and listen to each other's stories, and in this interaction, the students understand the patient's life‐world in a new and deeper way. By moving between the students’ understanding of caring theory and their own and others’ experiences, the students gain new perspectives on how to be caring. *The caring theory has shown me how important it is to do things with caring*. *It is almost impossible to do things without using caring*. *Caring goes into everything*, *and it is important that you use it no matter what you do as a nurse and who you talk to*. Thus, the caring theory has a possibility to change nurse students as human beings, and alter their thinking, actions, doing, and being.

### Reflection contributes to increased self‐understanding and awareness of oneself as a caring nurse

Reflection allows nurse students to get to know themselves better. Through reflection together with others the students share their feelings, thoughts and in this inner process the students become aware of their own understanding and opinions. *During and after the reflection groups*, *we get to think about philosophical questions to which there is not necessarily a definitive answer*. *But it requires that we must go into ourselves to ponder*, *and thus become better acquainted with ourselves and our opinions*. By sharing experiences and opinions, they become more aware of who they are and what influences their perception of other people and situations. At the same time, they become more aware of how they affect other people with their personalities. *By sharing experiences and views with other fellow students*, *we can expand our insight and create awareness within ourselves*, *in meetings with patients and relatives*. Reflection gives an increased awareness for assessing oneself. In the reflection, the students take an "outside look" at themselves and try to look at themselves as others see and experience them. By seeing themselves in this way, they become more reflective and aware of themselves. *There may have been things that were inside me before*, *but now I am much more reflective and aware of the choices I make*, *the way I speak*, *and the way I act*. In reflection, students obtain new ideas about how they want to become caring nurses, and how to develop their caring attitude. They received many new thoughts on how to behave as a caring nurse. *You need to be aware of yourself*, *have good self*‐*awareness*, *and think about what you say and do*. This understanding makes them more self‐aware, and they become better able to assess the quality of their caring behaviors. *I think it will be easier to think and reflect on what I do well and what can be improved in practice after we have discussed the concept of caring in the reflection*. Thus, the reflection grounded in caring theory facilitated the understanding of self, both professionally and personally.

Reflection offers new thoughts about one's desire to meet the patient in a caring way. Reflection initiates an inner process of change, where the students become more aware of how they treat and relate to other people. *The reflection groups have made me more aware of how I am towards patients and other people when I encounter them in different situations in everyday life*. *As well as how to act in different situations*. By reflecting on who they are and how they appear in meeting other people, they discover who they are and who they want to become and develop reflective abilities that they can use as a tool in the learning process of becoming the caring nurse they want to become. *I think this reflection is incredibly useful in the job of becoming the best possible nurse*, *and the best version of oneself*, *that one can become*.

## DISCUSSION

The results show that students participating in reflection groups and reflection grounded in caring theory is a valuable method for the nursing student's appropriation of caring theory. The appropriation of caring theory provides a starting point for their professional and personal formation to becoming a caring nurse. This is in line with Ekebergh's [35, 36, 42] and Lindberg et al.’s [26] statement that didactics that enable embodied reflection from a life‐world perspective in combination with caring science theory, and didactics that create relationships that enable shared learning can strengthen the students’ learning process so that they can gain a deeper understanding of caring and caring science.

The students described that the caring theory was unknown and difficult to understand. Yet, they demonstrated a willingness to challenge their previous understanding, and together with their fellow students, the caring theory was reflected on and intertwined with earlier knowledge. The students expressed that the encounter with the caring theory, challenged their previous understanding, and in their reflection, they realise and understood something new about caring and themselves in terms of becoming a caring nurse. By developing a language for caring in nursing, the students moved from struggling to understand to an understanding in which the caring concepts are embodied. This is in accordance with Lindström [43], who argues that when the caring theory is incorporated into previous understanding, it becomes part of one's own personal bearing, and the students’ previous understanding and level of development evolve to a deepened understanding and personal growth. This can be compared with the process of the professional development of a professional identity [44].

According to the students, through the movement towards a deeper understanding of caring and caring theory they become aware of their ethical stance. Ethos is an important foundation for becoming and formation [7]. Ethics and ethos represent a bearing, that is, a stance, and are regarded as the caregiver`s innermost core. This leads to a deepening of understanding that one's own caring attitude influences one's thinking, actions, and being. Through the embodied reflection in the relationship with the other, students became more aware that their professional caring identity is important for being able to face patients’ suffering and appeals for help. These findings agree with Hörberg [22] showing that true caring is not possible without incorporating a caring attitude and constantly reflecting on the care given and how it is received by patients. Through the dedication of caring theory, the students take a new step forward towards forming a caring identity, and a new base to stand on and undergo formation. This movement could be described as a hermeneutical spiral consisting of interconnected loops taking the students further and deeper into their process of understanding caring and professional formation to becoming a caring nurse, which is also described by Sandvik et al [2] and show that formation is a molding process where understanding requires formation, while in turn, formation is a prerequisite for understanding and appropriation. This leads to the formation and is the result of an ongoing internal process, where the appropriating of caring science theory provides a foundation for professional identities and ethical awareness [29].

The findings show that the effect of students’ appropriating of caring theory is about creating an understanding of themselves. Through the appropriating of the caring theory, the students learn about themselves, their own knowledge and experiences, and perceptions of the responses of others. According to the students, they used their own and each other's past experiences and pre‐understanding, also described by Knutsson et al. [45] as the natural stance, as a starting point to create a deeper understanding of caring and of themselves as caring nurses. These findings resonate with Sandvik et al. [2, 46] and Ekebergh [32] showing that reflection sets students’ inner processes in motion. Students explained that, through the reflection process, they became more open‐minded, wondering, and questioning in their meetings with fellow students’ reflections on the caring theory. From a phenomenological point of view, there is a great potential in learning together as we need others to know ourselves and vice versa [47] However, it can be difficult for students to challenge their natural attitude and the unreflective attitude. We found that students were open and honest about their own prejudices and vulnerabilities. According to the students, reflection with others facilitates the student's self‐reflection, and through self‐reflection, the students become aware of their own values and views on existence and life. This can be compared with Bengtsson [48] and Ekebergh [32], who reveal that in the theoretical context of nursing education, a conscious and active self‐reflection enables students to discover themselves in the light of theoretical caring science knowledge. Simultaneously, through appropriation, the caring theory becomes a ground for developing self‐awareness and self‐understanding, and a starting point for growth, both professional and personal.

## CONCLUSION AND IMPLICATIONS

This study provides further understanding of how participation in reflection groups where the reflection is grounded in caring theory, provides a foundation for the nurse students to see themselves within a broader perspective and how significant mutual support is for students’ professional formation and becoming a caring nurse. The study shows that when the students understand and internalise caring theory, the caring theory can be translated into a clinical situation and integrated into their way of knowing, acting, and being. Consequently, the appropriated caring theory provides an important starting point for their formation and becoming a professional caring nurse. The result shows how instruction in caring theories, participation in reflection groups, and reflecting on key concepts from caring theories have a key function in facilitating students’ development of a language for caring in nursing, provides wider understanding for human beings as a patient, and increased self‐understanding for oneself as a human being and caregiver.

Professional nursing care and caring are highly dependent on teaching and learning transactions starting from the academic setting and the theoretical standpoints. The better students understand the connection between caring as theory and practice based on teaching and reflection, the more the appropriated knowledge and attitudes will be transferred to clinical practice environments. The expected outcome of the integration of theory and practice in the nursing curriculum thereby adopt to progressively supports the development of nursing students both personally and professionally in their formation of becoming caring nurses.

## METHODOLOGICAL CONSIDERATIONS AND STUDY LIMITATIONS

The study had a qualitative design employing a thematic analysis [40] which was assessed as suitable for the character of the research subject. Individually written self‐reflection was performed at one university in Norway involving 64 undergraduate nursing students in their first term. The number of participants can be valued as quite small, but after reading the material we found that the quality was sufficiently large and varied to obtain information power to develop new knowledge, referring to the aim of the study [49]. That the participants came from the same university can be seen as a strength, although they had received the same instruction in caring theory and participated in the same reflection groups. The reflection groups were led by numerous teachers which can be seen as a weakness. Still, this can be a strength in producing more nuanced and richer data. The material consists of 44 typed pages, which may be considered a weakness, but they were rich in content. The written self‐reflections have been shaped by the participants writing something that they know will be read by the researcher, which may be considered a weakness, but because the purpose was not to investigate what the students had learned about caring and caring theory, but to gain knowledge about the students’ own reflections in relation to themselves in becoming caring nurses, this can be seen as a strength in producing more nuanced and richer data.

## CONFLICT OF INTEREST

The authors declare that there is no conflict of interest.

## AUTHOR CONTRIBUTIONS

The first author has been responsible for the research design, data collection, data analysis, and writing of the article. The second and third author has acted as supervisors and participated in the analysis and completion of the article.
